# Investigating the physiological role of S199A and S199D mutants of PHF6 protein in T-cell acute lymphoblastic leukemia

**DOI:** 10.55730/1300-0144.5689

**Published:** 2023-08-11

**Authors:** Gökçe ERDOĞAN, Osman Nidai ÖZEŞ, Alphan KÜPESİZ, Şükran Burçak YOLDAŞ

**Affiliations:** 1 Department of Medical Biology and Genetics, Faculty of Medicine, Akdeniz University, Antalya, Turkiye; 2 Department of Pediatrics, Faculty of Medicine, Akdeniz University, Antalya, Turkiye

**Keywords:** PHF6, T-cell acute lymphoblastic leukemia, NOTCH1, phosphorylation, mutation

## Abstract

**Background/aim:**

T-cell acute lymphoblastic leukemia (T-ALL) is a form of leukemia characterized by the proliferation of immature T lymphocytes. NOTCH1 is one of the most frequently mutated genes in T-ALL. NOTCH1 expression in T-cell development depends on plant homeodomain finger protein 6 (PHF6), which plays a tumor suppressor role in T-ALL. Several studies have shown that PHF6 expression is essential for NOTCH1 expression. Therefore, whether posttranslational modification of PHF6 plays a role in the regulation of NOTCH1 expression and T-ALL cell line proliferation was investigated herein.

**Materials and methods:**

The amino acid sequence of PHF6 was analyzed and it was found that a putative protein kinase A (PKA) phosphorylation motif RDRS199 was conserved in several vertebrate species and the S199 site was expected to be phosphorylated according to the PhosphoSite database. Therefore, an eukaryotic expression vector of human PHF6 was constructed, and the codon 199 was changed to the codon encoding the nonphosphorylatable alanine and the phosphorylation-mimicking aspartic acid via site-directed mutagenesis. After confirming the ectopic expressions of the PHF6 vectors by western blot analysis, the effects of these proteins were identified on the NOTCH1 expression using western blot analysis, leukemic cell proliferation using MTT assay, and expressions of the cell surface markers of T-cells using flow cytometry.

**Results:**

The ectopic expression of wild-type PHF6 stimulated the formation of CD4 + T-cells. While the expression of the wild-type PHF6 suppressed the growth of the leukemic cell line, this effect was diminished in both the alanine and aspartic acid mutants of PHF6. In addition, both mutants also seemed to negatively affect the NOTCH1 expression, although the effect of the alanine mutant was more severe.

**Conclusion:**

Taken together, the different biological activities exerted by the conserved S199 phosphorylation-site mutants shown in this study implicate that signaling pathway(s) leading to differential phosphorylation of this residue may have a substantial effect on the activity of PHF6, and thus may constitute a potential therapeutic target in T-ALL.

## 1. Introduction

**Figure 1:**
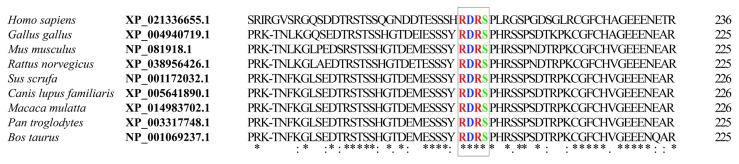
Conserved partial sequence of PHF6 containing the RDRS199 motif (colored in box) in several vertebrate species. PHF6 protein sequences were oriented with the Clustal Omega multiple sequence alignment tool for comparison.

The plant homeodomain finger protein 6 (PHF6) gene (gene ID: 84295) has a 1095 basepair (bp) cDNA. The PHF6 gene encodes a 41290 Da zinc-finger protein containing 365 amino acids (uniprot: Q8IWS0) [1]. Somatic loss-of-function mutations of PHF6 are most commonly seen in T-cell acute lymphoblastic leukemia (T-ALL) (15% of pediatric cases, 35% of adult cases) and wild-type PHF6 is a known tumor suppressor in T-ALL [2]. PHF6 has 2 atypical plant homeodomains (PHD) and mutations causing T-ALL are most commonly seen in the extended PHD (ePHD) domain [3]. As given in Figure 1, the RDRS phosphorylation motif located between the 2 PHD domains is conserved across many vertebrate species. PHF6 plays a role in hematopoiesis, brain development, and DNA damage repair [4–6]. It is a chromatin regulator and performs this activity by binding to the nucleosome remodelling and deacetylase complex, thereby regulating the transcriptional activity of some genes involved in hematopoiesis and oncogenesis [7, 8]. Like PHF6, activated NOTCH1 has been reported to interact with the nucleosome remodeling and deacetylase (NuRD) complex in T-ALL [9]. The highest expression of PHF6 is seen in double-positive (DP) T-cells and CD3 + CD4 + CD8 − T-cells; however, the mechanism of action of PHF6 in T-cell differentiation and proliferation is not fully understood [10,11]. NOTCH1 plays a key role in hematopoiesis and early T-cell differentiation. Hyperactive NOTCH1, with a gain-of-function mutation, is responsible for 50%–60% of T-ALL prognoses [12–14]. It has been shown in different studies that NOTCH1 mutation was also found in cases with PHF6 gene mutation and the NOTCH1 expression decreased in cells via the suppression of PHF6 [7,9,11,15,18].

Moreover, the loss of PHF6 results in a reduced expression of NOTCH1, which accelerates T-cell development. These results imply that PHF6 positively affects the expression of NOTCH1 and seems to play a significant role in normal human hematopoiesis. These results also strongly suggest the possible involvement of PHF6 in the development of T-ALL [9,19]. Supporting these results, Todd et al. also mentioned that NOTCH1 and PHF6 were coexpressed and PHF6 was bound to the promoter of NOTCH1, possibly inducing NOTCH1 expression [17]. As PHF6 seems to be an important regulator of NOTCH1 expression, any cellular event regulating PHF6 function should have an influence on NOTCH1-mediated T-cell development, and therefore, T-cell leukemic blast formation. To our knowledge, phosphorylation is one of the main signaling mechanisms to regulate a transcription factor function in a reversible manner. Therefore, it was hypothesized that PHF6 phosphorylation could regulate intracellular NOTCH1 levels, reduce T-cell proliferation, and direct DP T-cells to differentiate into either CD4 + or CD8 + T-cells.

## 2. Materials and methods

### 2.1. Study design

**Figure 2:**
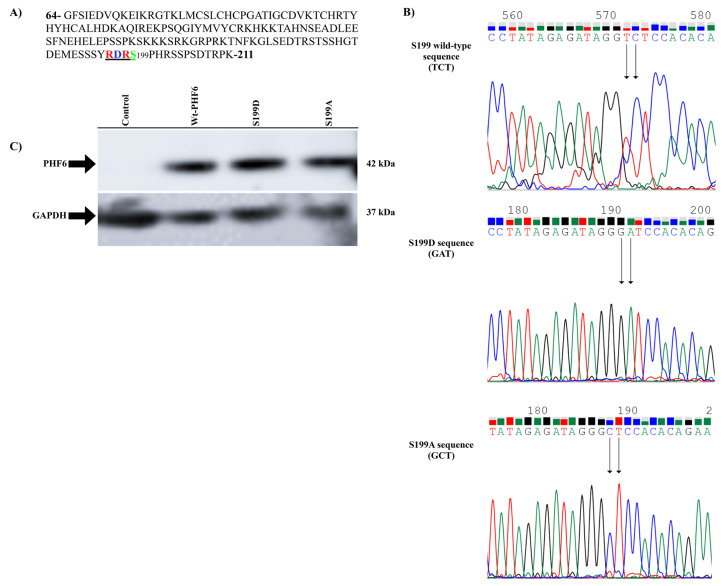
RDRS199 motif of PHF6 is underlined (a). Nucleotide sequences of codon 199 in wild type, aspartic acid, and alanine mutants of PHF6. Changed nucleotides are shown by the black arrow. The sequence analysis was performed using an Applied Biosystems 3130XL genetic analyzer via DNA Sequencing Analysis Software v5.1 (b). PHF6wt and mutant vectors can be ectopically expressed in DND41 cells (c).

First, the primer design for the site-directed mutagenesis of the wild-type and mutant PHF6 expression vector was constructed using the pcDNA3.1A plasmid. There were 3 primer pairs designed for the alanine, aspartic acid, and wild-type serine codons at position 199 of the PHF6 protein, as seen in Figure 2. After performing the site-directed mutagenesis, the DND41 cell line was cultured and duplicated for the leukemic cell experiments. PHF6 expression vectors were transfected into the DND41 cells, and protein lysates were collected for western blotting while at the same time performing MTT cell proliferation assay and flow cytometry with the same passage of cells. This was all performed at the Akdeniz University, Department of Medical Biology and Genetics laboratory, from May 2018 to April 2022.

### 2.2. Antibodies and reagents

Monoclonal primary antibody for anti-PHF6 (STJ29530) was purchased from St John’s Laboratory (London, UK). The monoclonal antibody for NOTCH1 (D6F11, 4380S) was from Cell Signaling Technology (Danvers, MA, USA) (NEB), and that for GAPDH (sc-47724) was from Santa Cruz Biotechnology (Dallas, TX, USA). The horseradish peroxidase (HRP) conjugated secondary antibodies to mouse and rabbit IgG were from Kirkegaard & Perry Lab Inc. (Gaithersburg, MD, USA) and Advansta (Menlo Park, CA), respectively (474-1806 and R-05072-500).

### 2.3. Plasmids and site-directed mutagenesis

In order to analyze the biological activity of PHF6 in the T-ALL cell line and its putative PKA phosphorylation motif, a eukaryotic PHF6 expression vector was created using the pcDNA3.1A plasmid and mutated the conserved Serine199 codon to the codons encoding alanine (A) and aspartic acid (D) via site-directed mutagenesis. The PHF6 wild-type (PHF6wt) expression plasmid was created by cloning the single open reading frame of PHF6 into the pcDNA3.1A backbone. Following validation of the vector efficiency by transfection into HEK293T cells, the procedure continued with site-directed mutagenesis, which was performed by Phusion polymerase (New England Biolabs, Beverly, MA, USA) reaction. First, 50 ng of plasmid DNA was used for the polymerase chain reaction (PCR) amplification in a 50-μL reaction volume. PCR conditions for the site-directed mutagenesis consisted of denaturation at 95 °C for 30 s, 16 cycles at 95 °C for 30 s, 55 °C for 1 min, and 72 °C for 14 min, with a final extension at 72 °C for 15 min, using a thermal cycler TurboCycler Lite (BlueRay Biotech, Taipei, Taiwan). PCR amplification was followed by DpnI enzyme (Molecular Biology; Thermo Fisher Scientific, Waltham, MA, USA) digestion at 37 °C for 15 min. Sequence PCR was performed by a denaturation step at 95 °C for 3 min, 30 cycles at 95 °C for 30 s, 70 °C for 30 s, 72 °C for 1 min, followed by 15 cycles at 95 °C for 30 s, 58 °C for 30 s, and 72 °C for 1 min, with a final extension at 72 °C for 15 min. PCR amplicons were purified with the NaAc/ethanol precipitation method. Cycle sequencing was performed using the BigDye Terminator v3.1 Cycle Sequencing Kit (Applied Biosystems, Foster City, CA, USA) at 96 °C for 1 min, 25 cycles at 96 °C for 10 s, 50 °C for 5 s, and 60 °C for 4 min. The S199 codons, as shown in Figure 2a, and plasmid sequences, given in Figure 2b, were validated by Sanger sequencing using an Applied Biosystems 3130XL Genetic Analyzer system (Thermo Fisher Scientific). The cloning and mutagenesis primers are shown in the Table.

### 2.4. Cell culture, transfections, and treatments

DND41, a T-ALL cell line with a truncated, therefore, nonfunctional PHF6, was obtained from DSMZ (Leibniz Institute DSMZ - German Collection of Microorganisms and Cell Cultures, Braunschweig, Germany). The cell line was cultured in RPMI-1640 medium (Invitrogen, Thermo Fisher Scientific) supplemented with 10% fetal bovine serum (FBS) (Life Technologies, Inc., Grand Island, NY, USA) and 1% penicillin G/streptomycin (Invitrogen, Thermo Fisher Scientific) at 37 °C in a humidified atmosphere under 5% CO_2_ cell culture environment. The lipofectamine (Invitrogen, Thermo Fisher Scientific) mediated transfection method was used for the transfections. Cells were transfected via reverse transfection protocol [20,21]. Briefly, in different experiments, 300 ng or 3 μg of plasmid DNAs were added into 25 μL or 250 μL of serum-free RPMI-1640 culture medium on microtiter plates and incubated at room temperature for a few minutes. Following this step, 0.4 μL (for 300 ng of DNA) or 8 μL (for 3 μg of DNA) of lipofectamine was added into 25 μL of serum-free RPMI-1640 medium, which was then transferred into the tubes containing DNA. The lipofectamine and DNA were incubated for 30 min at room temperature to allow the lipofectamine/DNA complex to be formed, which was then added to the cells. The medium was replaced every 3 to 4 days.

### 2.5. Western blot analysis

The radioimmunoprecipitation assay (RIPA) buffer (Sigma-Aldrich, St. Louis, MO, USA) was used to extract proteins from the cells. The cells were seeded at a density of 1 × 10^6^ cells/well on a 6-well microtiter plate by transfection, and then incubated overnight. The next day, the cells were centrifuged and washed with 1X phosphate buffered saline (PBS) and this step was repeated 3 times. They were then incubated on ice with the RIPA buffer supplemented with a protease inhibitor tablet (Pierce Biotechnology, Inc., Thermo Fisher Scientific). Following this step, the cells were collected into tubes with a cell scraper and used in the western blot analysis. Protein concentrations were obtained using the Bradford method. Protein lysates between 50 μg and 100 μg were homogenized in 2X sodium dodecyl sulfate (SDS) sample loading buffer, incubated at 95 °C for 4 min, and then placed on ice for 1 min. Samples were, then, loaded directly onto 7% or 10% 2-dimensional SDS-polyacrylamide gel using the Mini Protean Tetra Cell system (Biorad, Hercules, CA) and Standard Electrophoresis Blotting System (Hoefer, MA, USA), and then transferred to immobilon-P polyvinylidene difluoride membrane overnight. The running voltage for the stacking gel was 40 V for the mini gel, and 120 V for the standard system; the separating voltage for the mini gel was 70 V, and it was 150 V for the standard system. Transfer conditions were 40 V at 4 °C overnight. The membrane was blocked by a blocking buffer containing 1X PBS, 0.1% Tween-20 in 1% bovine serum albumin (BSA) (1 × PBS-T in 1% BSA) for 2 h. The blots were labeled with primary antibody (antirabbit PHF6 at 1:1000 dilution, antimouse GAPDH at 1:10000, or antirabbit NOTCH1 at 1:500) for 1 h at room temperature or overnight at 4 °C, respectively. Next, the blots were washed for 2 × 30 min with 1 × PBS-T to remove unbound antibodies, then labeled with the corresponding HRP-conjugated secondary antibody (antirabbit at 1:5000 or 1:25000 dilution, antimouse 1:25000) at room temperature. Then, the blots were washed for 3 × 10 min with 1 × PBS-T to remove unbound antibodies. Proteins were visualized with western bright enhanced chemiluminescence HRP substrate (K-12045-D50; Advansta). All of the antibodies were diluted by 0.1% Tween-20 in 1% BSA.

### 2.6. Flow cytometry

The effect of PHF6 on the CD4/CD8 T-cell differentiation was evaluated using flow cytometry. The monoclonal antibodies were purchased from BD Biosciences (Franklin Lakes, NJ, USA) and comprised CD1a APC (559775), CD2 PE-Y7 (335821), CD3 V-450 (558117), CD4-PECP 515 (332772), CD7 PE (332774), and CD8 APC-H7 (641400), and the TdT-FITC (F7139) was from Dako Denmark (Glostrup, Hovedstaden, Denmark). The DND41 cells were analyzed for T-cell surface antigens CD1a, CD2, CD3, CD4, CD7, CD8, and TdT in accordance with the current guidelines for the classification of leukemia by flow cytometry [22,23]. Next, 2 × 10^4^ cells per 1 μL were counted for analysis. One group of cells was transfected with the wild-type PHF6 expression plasmid with lipofectamine, while the control group was not transfected. After 24 h of incubation at 37 °C, the cells were centrifuged at room temperature, and the cell pellets were resuspended in 1 mL of FBS and 4 mL of 1 × PBS. The cells were then labeled with anti-CD2, CD1a, CD3, CD4, CD7, CD8, and TdT antibodies. Flow cytometric analysis was performed according to the manufacturer’s instructions using BD FACSDiva 7.0 software (Becton Dickinson, Mississauga, ON, Canada) in the BD Facs Canto II platform (Indianapolis, IN, USA).

### 2.7. Cell proliferation assay with MTT

The effects of the PHF6 wild-type and mutant vectors on leukemic cell proliferation were assessed via 3-(4,5-dimethylthiazol-2-yl)-2,5-diphenyltetrazolium bromide (MTT) cell proliferation assay. The MTT protocol was applied as Ersöz and Adan [24] had reported previously. Briefly, MTT was prepared as a stock solution (5 mg/mL) in 1 × PBS and filter-sterilized with a 0.22-μm filter [24]. Then, 5 × 10^4^ cells were transfected with mock or expression vectors of the wild-type and mutant PHF6 proteins in 96-well microtiter plates by reverse transfection with 6 repetitions. At 48 and 72 h posttransfection, 20 μL of MTT solution was added into the wells and incubated in the dark for 3 h. After centrifugation, the medium was removed and 100 μL of DMSO was added to each well to dissolve the formazan crystals by gently shaking the plate for 5 min. The absorbance of the developed color was determined at 570 nm.

### 2.8. Statistical analysis

Statistical analysis of the MTT assay was performed with GraphPad Prism 9.3.0 (Boston, MA, USA) software, and the data were presented as the standard error of the mean (SEM). PHF6 nontransfected DND41 mock cells were used as the control group. For analysis of the MTT assay data, the 1-way analysis of variance (ANOVA) (nonparametric) test was used, and parameters were compared using the Kruskal-Wallis test. Western blotting test results were analyzed with Image J software, and sequence results were analyzed with Chromas (South Brisbane, Australia) and SnapGene Viewer software (GSL Biotech; available at www.snapgene.com).

## 3. Results

### 3.1. The conserved serine-phosphorylation motif of PHF6

In order to find out whether there are any conserved phosphorylation sites on PHF6, the PHF6 sequence was scanned both by eye for known phosphorylation motifs and using the Phosphosite database. According to the proteomic data on the Phosphosite database, there are several phosphorylation, sumoylation, acetylation, and ubiquitination motifs on PHF6, as seen in Figure S1 [25]. These phosphorylations can cause conformational changes in the PHF6 protein and may lead to activation of the apoptotic pathway or regulation of proliferation or cell differentiation. Among these, S199 was the site to be phosphorylated in the 3 given organisms (human, mouse, and rat). S199 is preceded by the RDR sequence, which fits in the RXXS/T motif and can be phosphorylated by either PKA or CamKII [26,27]. When the corresponding sequence was analyzed via multiple alignment tools like the Clustal Omega multiple sequence alignment tool, it was observed that this sequence was conserved among several vertebrate species (Figure 1), which possibly implies its evolutionary importance [25,28]. Therefore, this site could be important for the biological roles of the PHF6 protein.

### 3.2. Expression of the wild-type PHF6 and its mutants in the leukemia cell line, and demonstration of the PHF6 mutants

In order to explore the functional significance of PHF6 S199 phosphorylation, PHF6 was first cloned into the pcDNA3.1A backbone, and then mutated Serine199 was cloned to the codon encoding alanine (A) to prevent phosphorylation and to the codon encoding aspartic acid (D) in order to mimic phosphorylation, via site-directed mutagenesis. Then, the vectors were transiently transfected into the leukemic DND41 cell line to show the ectopic expression of the wild-type protein and its mutants. To provide the ectopic expression of the wild-type and mutant PHF6 vectors, 3 μg of each vector was separately transfected into the PHF6-truncated DND41 cells using lipofectamine. Next, 48 h after transfection, the cell lysates were prepared in lysis buffer and equal amounts of protein were used for western blotting. After labeling the blot with an anti-PHF6 antibody, the same blot was labeled with an anti-GAPDH antibody to normalize the expression levels. As shown in Figure 2c, all of the proteins were expressed at very similar levels.

### 3.3. Effect of the PHF6 mutants on the NOTCH1 expression

**Figure 3:**
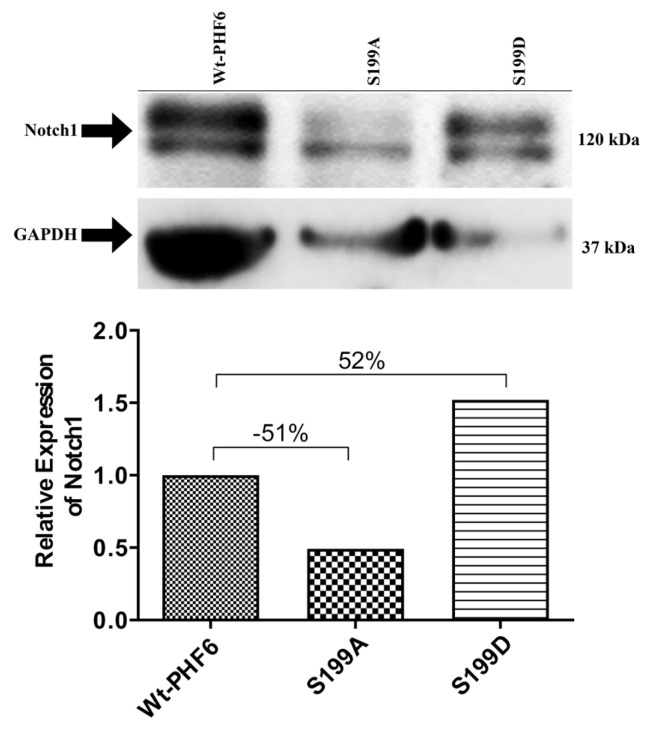
Effects of the wild-type and mutant PHF6 expression vectors on the NOTCH1 expression levels in the DND41 cells. Quantitative analysis was evaluated by normalizing to the NOTCH1/GAPDH levels.

As PHF6 is known to regulate NOTCH1 expression, we next questioned how PHF6 mutants influence NOTCH1 expression and how these compare to the wild-type PHF6. As shown in Figure 3, the ectopic expression of the wild-type PHF6 and its alanine and aspartic acid mutants differently affected the NOTCH1 expression. While the ectopic expression of the S199D mutant resulted in the highest level of NOTCH1 expression (52% increase), the S199A mutant suppressed the NOTCH1 expression in relation to the wild-type PHF6-transfected cells (51% decrease).

### 3.4. PHF6 mutants differentially affect cell proliferation

**Figure 4:**
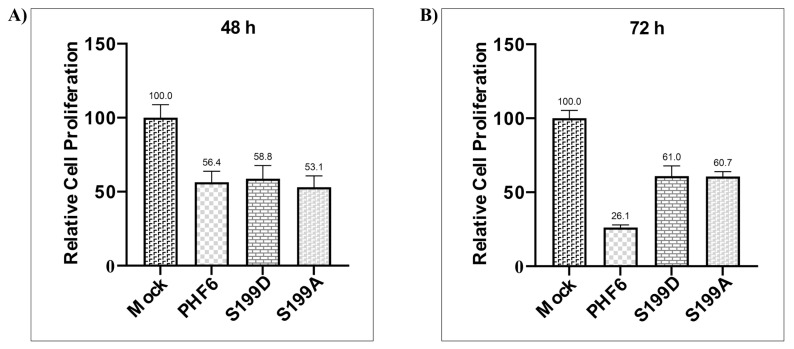
Effects of the wild-type and mutants of PHF6 on cell proliferation at 48 h (a) and 72 h (b) posttransfection. Cell proliferation was determined via MTT assay. Relative cell proliferations of the vectors are shown as percentages.

Since the S199A mutant of PHF6 significantly reduced NOTCH1 level and NOTCH1 is necessary for the proliferation of leukemic cells, the impact of wild-type PHF6 and phosphorylation mutants on the proliferation of DND41 cells was evaluated next. As presented in Figure 4, according to the MTT results, while the wild-type PHF6 did not significantly affect the proliferation of cells at 48 h compared to the mock control, as given in Figure 4a, it decreased proliferation at 72 h compared to the mock control, as given in Figure 4b. However, both mutants showed a much smaller effect on the suppression of cell proliferation.

### 3.5. Effects of PHF6 and its mutants on the cell surface markers of the T-cells

**Figure 5:**
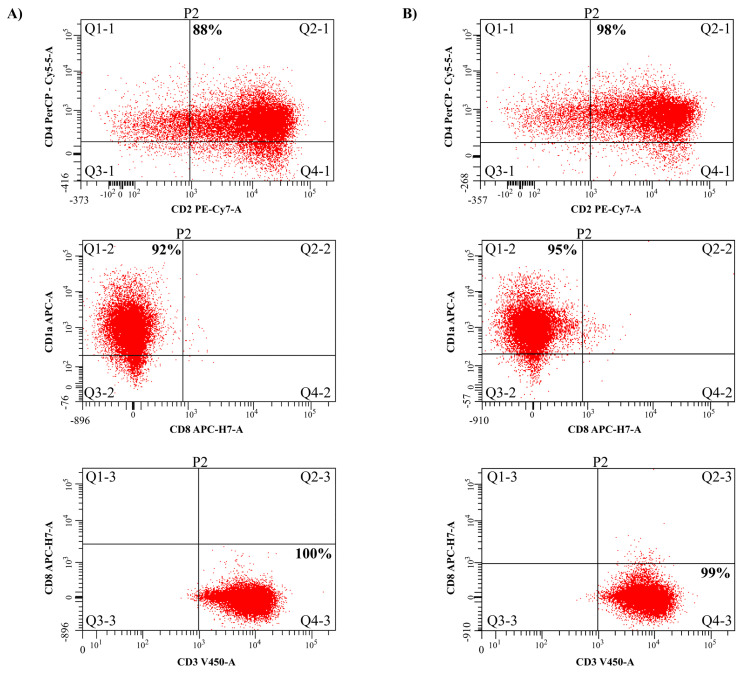
Analysis of the effects of PHF6 on T-cell differentiation for the surface antigens by flow cytometry. Flow cytometry output of the PHF6 negative control cells (a). Flow cytometry output of the PHF6wt cells (b). Both graphs show positivity for CD4, CD2, CD1a, and CD3, and negativity for CD8.

As the PHF6 expression was high in both the CD4 + CD8 + DP cells or CD4 + CD8 - cells, whether the PHF6 posttranslational modifications would also play a role in T-cell differentiation was explored by regulating the CD4 and CD8 antigen expressions. As seen in Figure 5, it was identified that the PHF6 control group cells were positive in CD1a, CD2, CD3, and CD4 antigens and these cells did not express CD8 antigen, as shown in Figure 5a. Furthermore, the PHF6wt cells were positive in the CD1a, CD2, CD3, and CD4 cell surface antigens, as shown in Figure 5b, and these cells were also CD8 negative. However, only a 1%–3% difference was observed between the cell surface markers, whereas a 10% increase in the CD4 + direction of the cells was observed in the cell group displaying the wild-type PHF6 ectopic expression.

### 3.6. Sequencing results

As shown in Figure S2, the quality values obtained from the ABI 3130XL instrument were as follows: all 3 of the sequencing data for the S199A, S199D, and PHF6 wild-type vectors had a high length of reading (LOR), with >500 bases (643, 733, and 777, respectively). Among these high LOR bases, the number of high-quality value (QV) bases were 572, 706, and 625 respectively. These results resulted in a high-quality score of 89% on average. Although all of the optimizations for the PHF6 wild-type vector sequence were used, the best peaks were from the forward primer sequence. Therefore, the image of the forward sequence of the region that was amplified was added, and the full sequence is shown in Figure S3. Although the wild-type Serine 199 (TCT) sequence had fluctuating peaks as it came to the end of the readout, the reliability and quality of the sequence confirmed the result.

## 4. Discussion

This study demonstrated that NOTCH1 in the DND41 leukemic cell line can be regulated by PHF6 S199 mutants. It also showed that ectopically expressed PHF6 can induce T-cells to form CD4 + T-cells.

PHF6, although expressed at its highest level in the brain tissue of mice as well as the thymus and ovaries of humans, is frequently associated with the development of T-ALL. In the last 2 decades, studies have shown that PHF6 plays a significant tumor suppressor role in T-cells and its mutation leads to T-ALL development [4,9,15,17,29]. Several studies have reported that PHF6 interacts with NOTCH1, one of the critical transcription factors in T-ALL oncogenesis, which has strengthened the idea that there is a feedback loop between these 2 molecules. NOTCH1 signaling plays a pivotal role in the development of T-cells and mutations in NOTCH1 are frequently associated with T-ALL. At the early stages of T-cell development, NOTCH1 induces proliferation, whereas in later stages, it induces the transcription of GATA3, thereby initiating CD4 + T-cell differentiation [30]. In T-ALL cells, NOTCH1 is coexpressed with PHF6 and PHF6 binds to the promoter of NOTCH1 [17].

In the current study, it was shown that the NOTCH1 expression could be modulated by PHF6 and its mutants. Since the PHF6 and NOTCH1 expression levels seemed to correlate in several cell lines and the DND41 cells were reported to have very low levels of NOTCH1, as indicated in the literature, it was decided to compare the NOTCH1 expression levels of S199-mutated PHF6-transfected DND41 cells with wild-type PHF6-transfected DND41 cells [16,31]. Surprisingly, compared to the ectopically expressed wild-type PHF6, the S199A mutants of PHF6 significantly reduced the expression of NOTCH1 while the S199D mutant displayed an inducing effect. Since the conversion of the phosphorylation target Serine199 to the nonphosphorylatable alanine had such a drastic effect on the NOTCH1 level, we strongly believe that the phosphorylation of S199, which was most likely mediated by the cAMP-activated kinase (protein kinase A (PKA)), alters the structure of PHF6 in such a way that the phosphorylated PHF6 can function as a tumor suppressor and induce the expression of NOTCH1. The findings herein were consistent with the literature [11].

In parallel with the findings of Loontiens et al., a higher expression of PHF6 was found in the CD4 + CD8− cells compared to the CD3 + CD4− CD8− cells in the current study, which strengthened the idea that PHF6 provides T-cell differentiation via the NOTCH1 pathway [11].

When the influence of the wild-type PHF6 and PHF6 mutants on the proliferation of the DND41 cell line, which lacked functional endogenous PHF6, was explored, higher proliferation was observed in both mutants compared to the ectopically expressed wild-type PHF6. Of note, both mutants exhibited lower cell proliferation compared to the untransfected mock cells. Thus, it might be suggested that the conformational change induced by mutating the S199 residue resulted in the suppression of some antiproliferative pathways, but not all [32]. As a direct influence of the S199D mutant on the induction of NOTCH1 expression was observed, these nonfunctional antiproliferative pathways should be independent of NOTCH1. Since the mock cells did not express a tumor suppressor gene, these cells proliferated more than 2-fold in comparison to the wild-type PHF6-transfected cells and increased 2-fold more in 72 h. There were some potential limitations of the current study. Herein, the impact of PHF6 and its S199D and S199A mutants on T-cell proliferation and differentiation was determined using the DND41 leukemic cell line, which lacks functional PHF6. Since it was only possible to investigate the influence on NOTCH1 expression, further research is needed to examine other T-cell marker genes. In addition, future studies are planned to demonstrate PKA-mediated PHF6 phosphorylation by in vitro kinase reactions and generation of PHF6S199A and PHF6S199D mutant animal models to further investigate the impact of PKA-mediated PHF6 phosphorylation.

In conclusion, the ectopic expression of mutant PHF6 vectors displayed different effects on leukemic cell proliferation, T-cell polarization, and NOTCH1 expression. The results suggest that S199 modification (phosphorylation) affects the activity of the PHF6 protein. In light of this, there may be an S199 motif-mediated linkage between NOTCH1 and PHF6 in terms of T-ALL pathology. While the results herein support the tumor-suppressive role of PHF6, they are in contrast to the oncogenic role that is attributed to this molecule in different cancer settings [8]. In future studies, phosphorylation of this motif can be evaluated in the leukemic cells of T-ALL patients, and if this transcription factor is hyperphosphorylated in T-ALL cells, animal studies will be informative, where an enzyme-inhibitor strategy is induced for treatment of T-ALL.

## Supplementary Information

Figure S1 Several posttranslational modification sites and conserved PKA kinase RDRS phosphorylation motif and S199 phosphorylation on PHF6, also obtained from the www.phosphosite.org database.

Figure S2 Sanger sequencing data summary for the S199A, S199D, and PHF6 wild-type plasmid sequences by ABI 3130XL genetic analyzer system software 6.

Figure S3 Full sequence analysis of the PHF6 wild-type vector via ABI 3130XL genetic analyzer system software 6.

## Figures and Tables

**Table:** Cloning and mutagenesis primer sequences of the PHF6 wild type, S199A, and S199D.

PHF6 vectors	Sequence
Wild type	Forward: 5′ - cgcggatccgccgccatgtcaagctcagttgaacagaaaaaaggg - 3′ Reverse: 5′ - cgcggatccgtttccattaagttgctgctgagttagttg - 3′
S199A	Forward: 5′ - tcctatagagatagggctccacacagaagcagc - 3′ Reverse: 5′ - gctgcttctgtgtggagccctatctctatagga - 3′
S199D	Forward: 5′ - tcctatagagatagggatccacacagaagcagc - 3′ Reverse: 5′ - gctgcttctgtgtggatccctatctctatagga - 3′
