# Supplementary figures and images for: Investigating the physiological role of S199A and S199D mutants of PHF6 protein in T-cell acute lymphoblastic leukemia
Source: Turk J Med Sci. 2023 Aug 11;53(5):1234–43. doi: 10.55730/1300-0144.5689 (PMC10763810; doi:10.55730/1300-0144.5689)

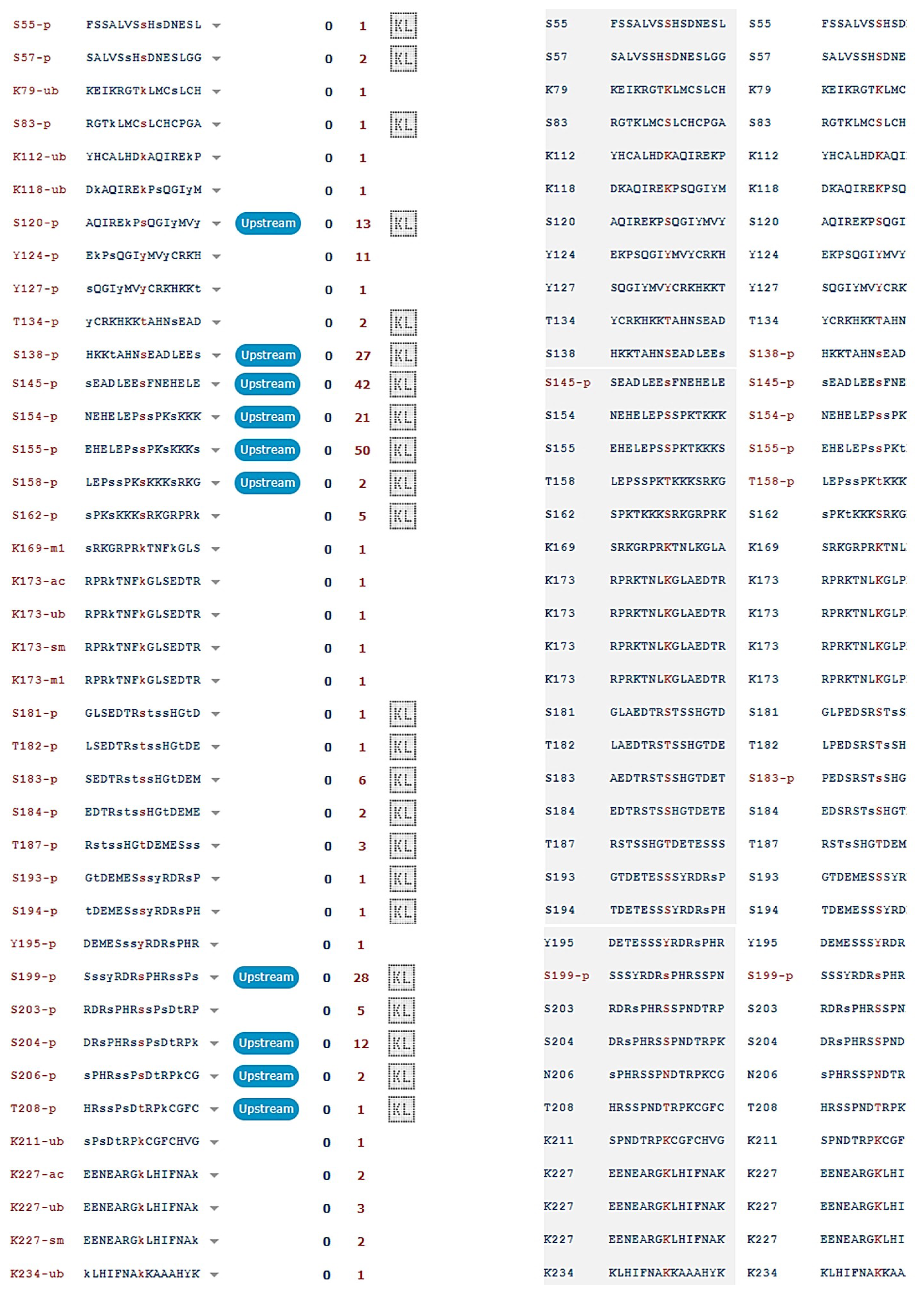

Supplement: Figure S1 — Several posttranslational modification sites and conserved PKA kinase RDRS phosphorylation motif and S199 phosphorylation on PHF6, also obtained from the www.phosphosite.org database. [file turkjmedsci-53-5-1234s1.tif]

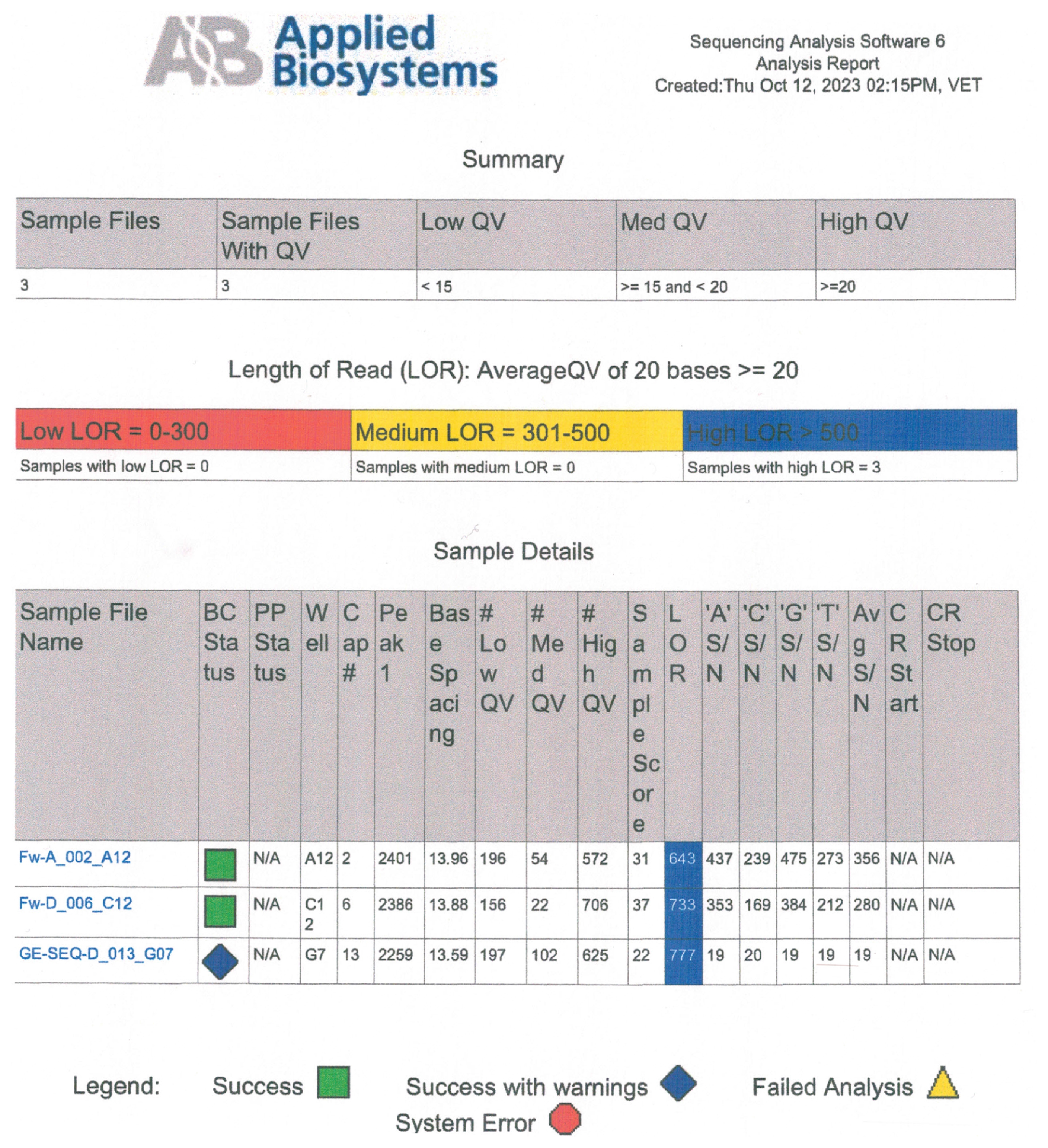

Supplement: Figure S2 — Sanger sequencing data summary for the S199A, S199D, and PHF6 wild-type plasmid sequences by ABI 3130XL genetic analyzer system software 6. [file turkjmedsci-53-5-1234s2.tif]

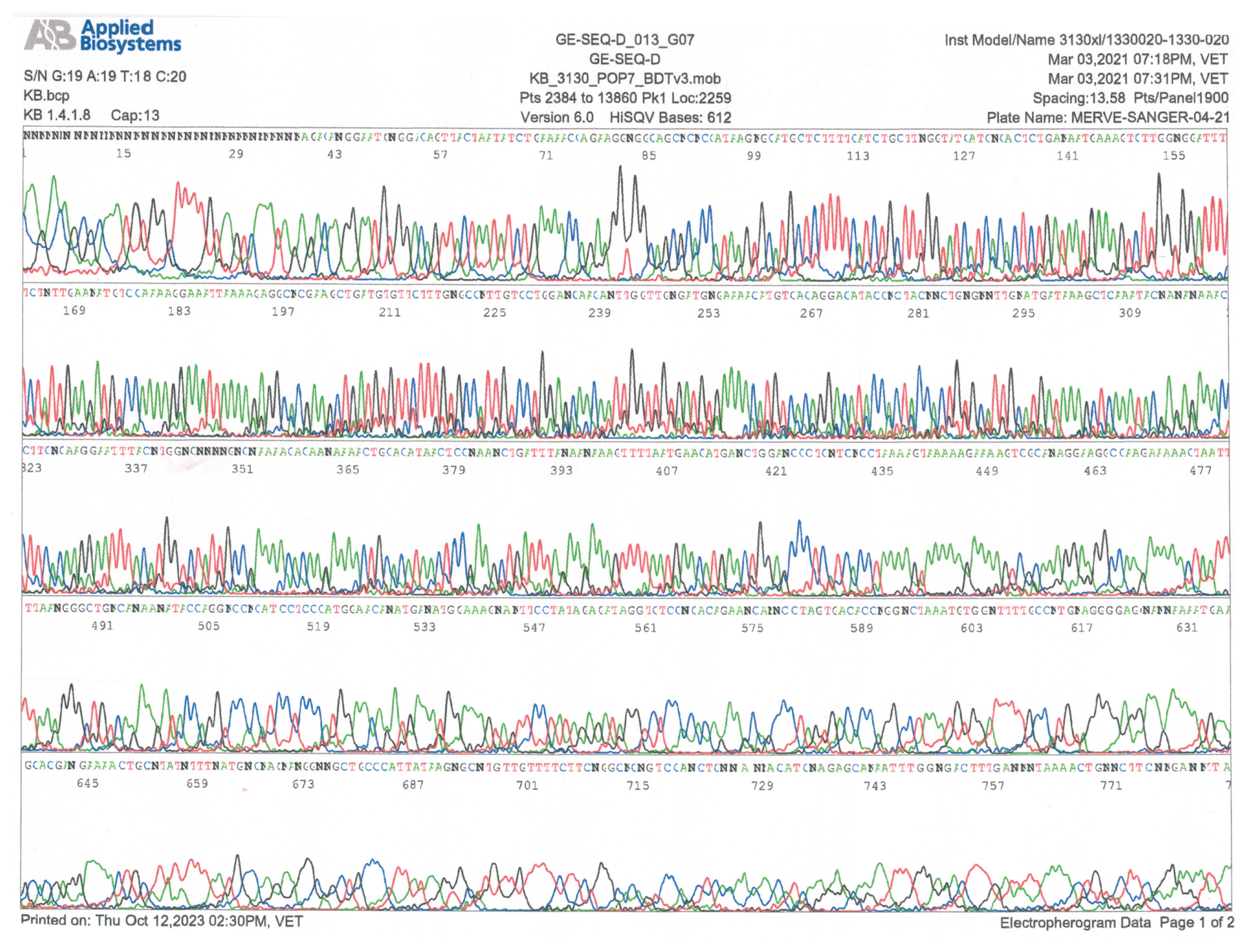

Supplement: Figure S3 — Full sequence analysis of the PHF6 wild-type vector via ABI 3130XL genetic analyzer system software 6. [file turkjmedsci-53-5-1234s3.tif]
